# A community-based system dynamics approach suggests solutions for improving healthy food access in a low-income urban environment

**DOI:** 10.1371/journal.pone.0216985

**Published:** 2019-05-14

**Authors:** Yeeli Mui, Ellis Ballard, Eli Lopatin, Rachel L. J. Thornton, Keshia M. Pollack Porter, Joel Gittelsohn

**Affiliations:** 1 Center for Human Nutrition, Department of International Health, Johns Hopkins Bloomberg School of Public Health, Baltimore, MD, United States of America; 2 Social System Design Lab, Brown School of Social Work, Washington University in St. Louis, St. Louis, MO, United States of America; 3 Reservoir Hill Improvement Council; Baltimore, MD, United States of America; 4 Center for Child and Community Health Research, Division of General Pediatrics and Adolescent Medicine, Department of Pediatrics, Johns Hopkins School of Medicine, Baltimore, MD, United States of America; 5 Department of Health Policy and Management, Institute for Health and Social Policy, Johns Hopkins Bloomberg School of Public Health, Baltimore, MD, United States of America; 6 Global Obesity Prevention Center (GOPC) at Johns Hopkins University, Baltimore, MD, United States of America; Indiana University Purdue University at Indianapolis, UNITED STATES

## Abstract

Little is known about the mechanisms through which neighborhood-level factors (e.g., social support, economic opportunity) relate to suboptimal availability of healthy foods in low-income urban communities. We engaged a diverse group of chain and local food outlet owners, residents, neighborhood organizations, and city agencies based in Baltimore, MD. Eighteen participants completed a series of exercises based on a set of pre-defined scripts through an interactive, iterative group model building process over a two-day community-based workshop. This process culminated in the development of causal loop diagrams, based on participants’ perspectives, illustrating the dynamic factors in an urban neighborhood food system. Synthesis of diagrams yielded 21 factors and their embedded feedback loops. Crime played a prominent role in several feedback loops within the neighborhood food system: contributing to healthy food being “risky food,” supporting unhealthy food stores, and severing social ties important for learning about healthy food. Findings shed light on a new framework for thinking about barriers related to healthy food access and pointed to potential new avenues for intervention, such as reducing neighborhood crime.

## Introduction

Prior research has shown variability in the food environment, eating behaviors, and obesity risk across neighborhood features, such as socioeconomic status and racial composition. For instance, low-income and minority communities tend to have less access to healthy food choices, poorer diet quality, and increased risk of obesity, compared to wealthier and predominantly White neighborhoods [1–4]. Questions remain about the mechanisms through which these and other neighborhood characteristics operate, in what ways they contribute to the landscape of food outlets in an area, and how best to support greater access to healthy foods in underserved neighborhoods. These research gaps are also reflected in a larger ongoing dialogue within public health about the need for new methods and tools to understand the interaction between multiple ecological levels within social systems, and to engage with feedback dynamics and emergent system behaviors. Rhetoric around the call for complex system approaches to public health has existed for years, yet concrete examples that provide actionable insight for policymakers or program implementers remain rare [5]. The promises of complex system methods include the ability to develop simulation models to explore relative leverage of policy interventions within dynamically complex systems such as urban food environments. In addition, these methods provide utility in formative stages of analysis, such as providing more nuanced conceptual models of problems that incorporate interaction between individual and community level factors; tools (formal models and simulation) to identify new potential leverage points or sources of policy resistance; and identification of novel hypotheses or data gaps to be filled through subsequent collection of empirical data [6]. Without insight into the causal links and feedback relationships embedded in neighborhood food systems, there is a risk that programs and policies may press on the wrong “levers,” or worse, result in adverse unintended consequences. For instance, while a tax on sugar-sweetened beverages may decrease the consumption of soft drinks and fruit drinks, it may also have the unintended consequences of increasing the consumption of fruit juices or promote product substitution with foods high in fat and sodium [7,8].

System dynamics is one approach for understanding and evaluating unintended consequences in complex systems that involve changing and evolving factors [9]. System dynamics uses qualitative causal maps and formal quantitative simulation models to explore and understand system behavior from a feedback perspective [10]. A type of qualitative causal map known as a causal loop diagram helps to visualize the factors in a system and their positive or negative relationships with one another using lines, arrows, and feedback loops. Causal loop diagrams are also used to critique and refine conceptual models of a given public health issue. Further, users can explore hypotheses about a system’s structure and identify potential intervention points that may lead to adverse or desirable behavior [10,11]. Causal loop diagrams are developed through a process called group model building, which is an engaging, iterative process that actively involves decision-makers, community partners, and trained modelers, to collaboratively model a complex problem [12]. Under the system dynamics umbrella, community-based system dynamics is an approach that focuses on building system dynamics models in partnership with community members embedded in these systems. What differentiates community based system dynamics from other group model building or participatory modeling approaches is the explicit focus on developing systems thinking capabilities among community members, including an endogenous or feedback perspective, appreciation for non-linear system behavior, and an emphasis on operational thinking [13]. The process results in qualitative models that have meaningful community input and builds new capabilities among community members to use such models for further dialogue and, ultimately, intervention development. Some investigators have employed community-based system dynamics to explore root causes of childhood obesity, while others have used this approach to identify key factors influencing health care utilization and service quality in destabilized communities [14,15].

Though it has been shown that access to nutritious foods is an important determinant of healthy living, little is known about the mechanisms through which the system of neighborhood-level factors might impact the landscape of food outlets and, consequently, food behaviors. Specifically, there is limited understanding of how neighborhood disorder–typically characterized by crime, vandalism, abandoned buildings, and other incivilities [16]–may create a context that influences the location of certain food outlets. Further, prior investigations have not leveraged community-based system dynamics to elicit perspectives from diverse stakeholders regarding the neighborhood system and barriers to healthy food access in an urban setting.

The purpose of this article is to describe the process and implementation of a group model building workshop with community members to: (1) learn about the range of factors (e.g., crime, employment, food stocking in stores, etc.) that affect the neighborhood food system and suboptimal healthy food access for residents; (2) explore different stakeholders’ perspectives on the nature and consequences of suboptimal healthy food access, and to develop common language between these groups to talk about these challenges; and (3) identify potential points of intervention to improve healthy food access.

## Methods

### Setting

The geographic region of interest for this study was Baltimore, MD, in particular the Penn North and Reservoir Hill communities in the 7^th^ District of Central West Baltimore. Nearly 80 percent of retail food stores in this District have very low Healthy Food Availability Index (HFAI) scores, and nearly 40 percent of residents live in areas lacking both vehicle ownership and sufficient economic resources to obtain healthy food [17]. In particular, Penn North and Reservoir Hill, a neighborhood with a HFAI score that is nearly two points lower than the City’s average at 7.7 (maximum of 28.5 points), leaves residents with poor access to fresh fruits, vegetables, and other healthful foods [18]. In 2010, volunteers from the Reservoir Hill Improvement Council Green Team came together to build Whitelock Community Farm on what was once a vacant lot in the neighborhood. While the Farm has continued to increase its growing capacity, sales, and programmatic efforts, healthy food access work continues to face challenges, namely in the less-resourced community of Penn North. To learn from neighborhood residents and partners about specific challenges they face regarding poor access to healthy foods, the Reservoir Hill Improvement Council hosted a mini Food Justice Conversation in August 2016. While insightful, participants were left unsure of the best strategy or leverage points to target in their food system to reduce barriers.

Given prior efforts in this neighborhood as well as community interest in intervention strategies, this group model building workshop focused on contributing to the ongoing efforts of Reservoir Hill Improvement Council and Whitelock Community Farm to improve healthy food access and inform strategic planning in Central West Baltimore.

### Recruitment

Leveraging existing networks between the Reservoir Hill Improvement Council and Whitelock Community Farm, and community members in Central West Baltimore, we used maximum variation purposive sampling to recruit 18 participants, representing a diverse group comprising: 3 chain and local storeowners, 8 community residents, 3 representatives from city government agencies, and 4 representatives from local non-profit organizations. Maximum variation purposive sampling is a technique used to document diverse variations in participants’ experiences, in this case related to healthy food access, and to identify common patterns [19]. The number of years participants had lived and worked in Baltimore City ranged from 0 to 75 years and 1 to 40 years, respectively. Individuals were between 25–64 years, with the exception of three who were 65 years or older. Participants were recruited from the Penn North and Reservoir Hill communities, with the exception of policymakers. Those who agreed to participate in the workshop were reimbursed with gift cards ($70 to Target) for their time.

### Design

Design of the group model building workshop was led by the core modeling team, which shared the responsibilities for the design, planning, recruitment, and facilitation of the workshop. This team included three members: lead researcher (YM); community partner (EL), and an expert consultant in group model building (EB). The workshop was held over the course of 2 days (2.5 hours each day) in December 2016. The research plan was deemed not human subjects research and exempted by the Johns Hopkins Institutional Review Board (IRB No. 4203) given that no personal or identifying information was collected (i.e., participants spoke only about their views and opinions in their professional/community role within their broader respective neighborhoods). As such, written participant consent was not collected for this study. Still, to ensure participants understood the nature of the study and were comfortable declining, we obtained verbal consent from each participant before beginning the workshop and to document the workshop with photographs and written notes. All participants verbally agreed to participate in the workshop, and again, due to IRB exemption, no record of verbal consent was collected. All data were analyzed anonymously.

### Scripts

Scripts are pre-determined, adaptable exercises used to facilitate group model building sessions. All scripts can be accessed online from *Scriptapedia* (https://en.wikibooks.org/wiki/Scriptapedia), which includes details on the procedures for executing each script, necessary materials, inputs, outputs, and more [12,20]. Six scripts were employed, and outputs from each step contributed to the next, until reaching the final output–a causal loop diagram that depicted participants’ perspectives of the interaction between factors that related to healthy food access in Central West Baltimore (Table 1).

**Table 1:** Description of the functions, activity, and outputs of each script employed in the group model building process.

Script name	Functions	Activity	Outputs
1. Hopes and fears	• To establish a set of values about embracing diverse perspectives and transparency of the process • Used for check-ins and informal evaluation throughout and after the session	Participants completed this activity individually, and then shared their contributions with the entire group.	List of participants’ hopes and fears
2. Behavior over time graphs	• To initiate mapping by generating multiple factors as potential drivers of the problem • To begin to generate rich narratives explaining the current community conditions, based on historical trends • To elicit hoped and feared trends for future system behavior	Participants completed this script in pairs, followed by discussion with the entire group.	Candidate factors for the causal loop diagram
3. Dots	• To sort through many possible choices and select those that are most important to the group	Each participant received 4 sticker dots to place next to any of the behavior graphs over time; participants completed this script individually, followed by discussion with the entire group.	Prioritized factors
4. Connection circles	• To introduce the concepts of causal connections and feedback relationships in a system • To make linkages between variables explicit	Factors discussed in Scripts #2–3 were used to develop connection circles. Participants completed this step in three groups, followed by discussion with the entire group.	1 connection circle per group
5. Causal loop diagrams	• To synthesize multiple perspectives of a problem and reveal new insights	Connections identified in Script #4 were expanded upon by including feedback loops and the identification of sub-systems and themes. Participants completed this step in the same groups as in Script #4, followed by discussion with the entire group.	1 causal loop diagram per group
6. Action ideas	• To identify action ideas and prioritize them along a matrix according to whether they are easy or hard to achieve (y-axis) and whether they would create low or high impact (x-axis)	Participants completed this step in the same groups as in Script #4 and 5, followed by discussion with the entire group.	Prioritized list of potential actions

The main objective of Scripts #1–3 was to elicit discussions on key factors that contribute to and are influenced by neighborhood disorder, the food environment, and food access. This was initiated around the question, “What affects access to fresh and affordable food in Central West Baltimore?” With Scripts #4–5, participants developed their causal loop diagrams to describe the interconnections and feedback relationships between these factors (S1 and S2 Figs), and using Script #6, together identified potential intervention points that could reduce food insecurity in Baltimore.

### Analysis

After the workshop and through an iterative process, the core modeling team synthesized the participants’ three causal loop diagrams into one system dynamics causal loop diagram, using Vensim PLE (version 6.4E). Supplemented by written notes documenting discussions from the workshop, the core modeling team also identified and named key themes and feedback loops. Each iteration of the causal loop diagram was compared against workshop notes and participants’ causal loop diagrams, to ensure that the final causal loop diagram accurately reflected participants’ narratives and causal logic shared in the workshop. Member checking was used to confirm or refine ongoing content analysis in a follow-up session with workshop participants who provided feedback on the synthesized causal loop diagram as well as on the themes and feedback loops that emerged.

## Results

**Fig 1:**
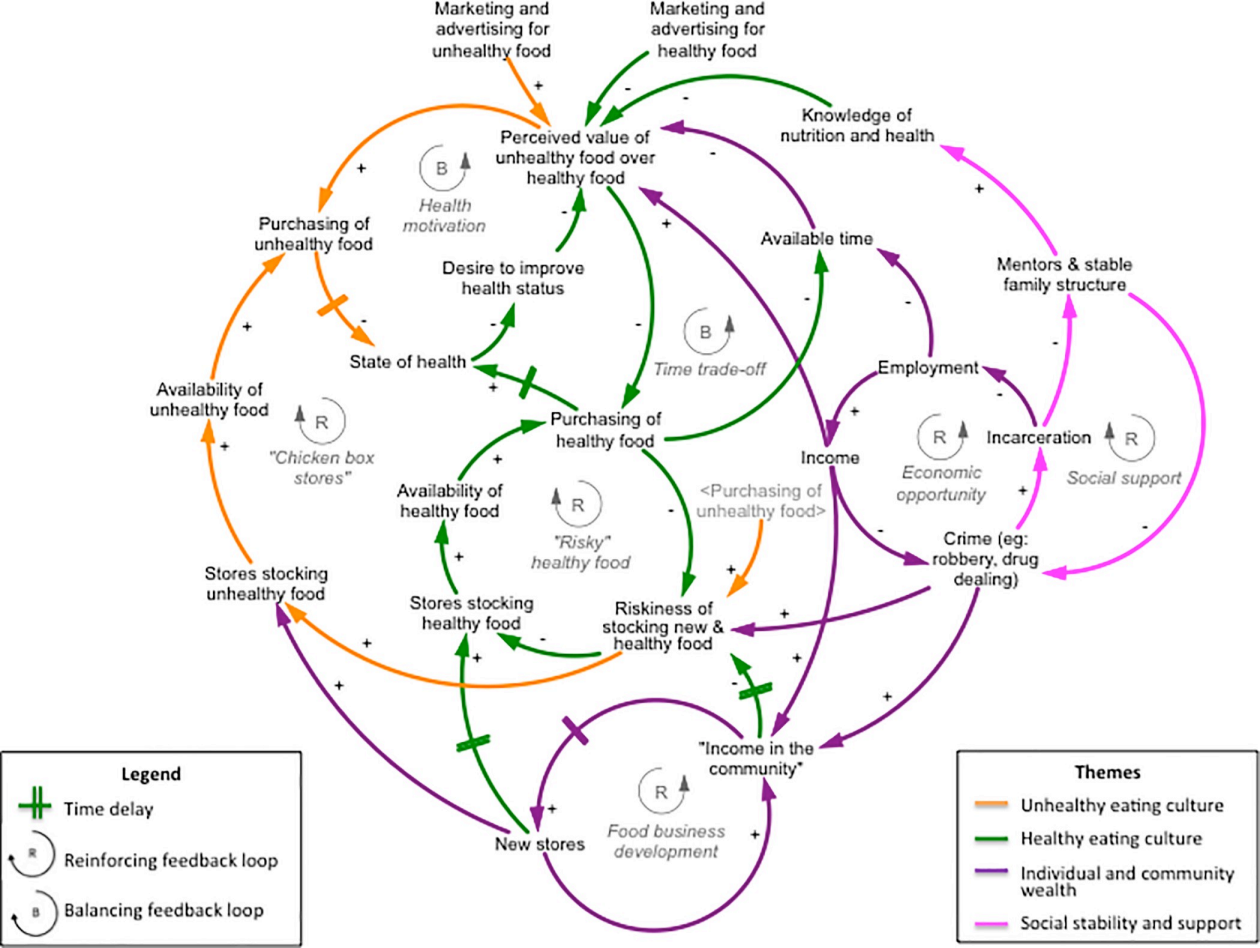
Themes and feedback loops in the synthesized causal loop diagram, initiated around the question, “What affects access to fresh and affordable food in Central West Baltimore?”*. *Solid lines with positive polarity (+) represent a relationship that can interpreted in two ways: increase in a factor causes an increase in the receiving factor, and a decrease in a factor causes a decrease in the receiving factor; solid lines with negative polarity (-) represent a relationship that can be interpreted in two ways: increase in a factor causes a decrease in the receiving factor, and a decrease in a factor causes an increase in the receiving factor.

The behavior over time graphs and connection circles scripts yielded 21 factors and their corresponding feedback loops that affect the neighborhood food system and access to fresh and affordable food in Central West Baltimore. Fig 1 is informed by diverse stakeholder perspectives and displays the major themes that emerged in the synthesized causal loop diagram, each designated by a different color: (1) Unhealthy eating culture (orange arrows); (2) Healthy eating culture (green arrows); (3) Individual and community wealth (purple arrows); and (4) Social stability and support (pink arrows). Key reinforcing feedback loops within these themes are described in further detail below and included: “chicken box stores,” “risky” healthy food, crime and food businesses, economic opportunity, and crime and social support. Balancing feedback loops, related to health motivation and time trade-off, are also explained in the following section.

### “Chicken box stores”

This reinforcing feedback loop in the unhealthy eating culture theme (orange arrows) indicated that greater availability of unhealthy food options (e.g., candies, chips, fried chicken) leads to a greater likelihood of purchasing unhealthy foods. Unhealthy food purchases reaffirm and increase storeowners’ perception that unhealthy food options are in greater demand than healthy options. In other words, as customers continue to purchase unhealthy foods, the perceived riskiness of stocking new and healthy foods increases. Thus, to meet this demand, storeowners maintain the stock of unhealthy foods, making them readily available and further promoting the purchase of those foods. Participants referred to these unhealthier food outlets (e.g., other carryouts and corner stores) as “chicken box stores.” One remarked, “All stores [are] selling the same thing in a community. Need to decrease things everyone is offering, like chicken boxes,” suggesting a greater variety of food outlets and food offerings in stores are needed to encourage healthier eating choices. Another mechanism through which the purchasing of unhealthy food can be offset is represented in the health motivation balancing loop. A participant explained that a decline in one’s health status motivates improving said health status, therefore, decreasing the perceived value and purchasing of unhealthy food.

### Healthy food is “risky food”

Reinforcing feedback loops in the healthy eating culture theme (green arrows) revealed that increases in the perceived value of unhealthy food over healthy food contribute to decreases in the purchasing of healthy food by consumers, which results in an increase in the perceived riskiness of stocking new and healthy food by storeowners. Higher levels of this perceived risk leads to less stocking of healthy food options in stores, contributing to an overall decreased level of healthy foods available. Participants linked less availability of healthy food options, compared to unhealthy food options, to a decreased likelihood of purchasing healthy food. Lower rates of healthy food purchasing, often due to the poor quality of limited healthy options, further perpetuates storeowners’ perceived level of risk of stocking new, healthy food options that are *seemingly* in low demand. Additionally, because many independently owned food outlets in this context operate on small profit margins, storeowners are careful to weigh the costs and benefits. Participants described crime (e.g., vandalism, robbery) directly increasing costs for storeowners in the form of enhanced security, restoration, or loss of property, which further increases the riskiness of stocking new and healthy food, as storeowners are less inclined to take on additional financial losses, should healthy food options not sell. A workshop participant stated, “People will not invest in higher priced items if they think they are going to get robbed.” In other words, for storeowners facing security concerns, the risk of business is already high. Therefore, from a business perspective, there is little incentive to stock a variety of healthy food options that would require a substantial investment by the storeowner. Decreases in the purchasing of healthy food, over time, can also result in the decline of one’s health status, therefore, activating another balancing loop related to health motivation, and decreasing the perceived value of unhealthy food.

### Crime and food businesses

Within the individual and community wealth theme (purple arrows), participants explained how crime, namely drug dealing, contributes to the community’s wealth or “income in the community.” One storeowner explained, “It’s true that those people selling drugs bring money not just for themselves, but for their families, so they can feed kids, grandkids. True [they] bring money to the community.” Participants further elaborated that in some cases, illicit drug dealers help “chicken box stores” with rent payments in exchange for a safe haven for drug operations (i.e., space where drug trade can occur that is not openly visible to the public and law enforcement). In other cases, drug dealers build “income in the community” as valuable customers. While individual-level characteristics, such as food preferences or time constraints, also influence store patronage, participants shed light on how crime, particularly in the form of illicit drug trading, is an important factor influencing “chicken box stores” and creating barriers for greater healthy food access. A storeowner said, “My family [has] been in that corner since ‘87, get to see now and then what’s been going on for last 30 years…I don’t think my wife is paying drug dealers any money, but she can’t put them out…Crime and food business[es] feed each other. If my wife didn’t have a profit off of all those drug dealers, she would have left there a long time ago.” Therefore, these co-dependent dynamics contribute to the viability of “chicken box stores” and, consequently, continued community access to the unhealthy food options typically stocked in the stores. One resident also shared, “If you take a drug dealer out, you may be causing a family to be hungry,” either the storeowner’s or drug dealer’s family, due to losing its main source of income through their respective businesses. Growth in employment opportunities also contributes to “income in the community,” which can promote the establishment of new food outlets. However, shifts in employment and new food outlets occur with a time delay (as indicated by the double lines across the arrow). With new stores come the possibilities of greater stocking of healthy food options, which could increase the availability and purchasing of healthy food, and decrease the perceived riskiness of stocking new and healthy foods in stores. However, the generation of new stores does not necessarily lead to the opening of new healthier food outlets, absent other concurrent changes in the food system.

### Economic opportunity

Reinforcing feedback loops in the individual and community wealth theme (purple arrows) revealed that lower income at the individual level was linked to a greater likelihood of criminal behavior, leading to incarceration and future unemployment, which in turn reinforces low-income status. Low-income individuals are price sensitive and unhealthier food options tend to be less expensive than healthier food options in this context. As the perceived value of unhealthy food over healthy food increases, this results in an increase in the purchasing of unhealthy food and a decrease in purchasing healthy food, which reinforces the healthy food is “risky food” feedback loop. Additionally, with an increase in employment, available time decreases, and the perceived value of unhealthy foods may also increase, given that healthier food options often require more time to prepare. With an increase in the perceived value of unhealthy foods, the purchasing of healthy food decreases, thus, freeing up time due to less food preparation and decreasing the perceived value of unhealthy food relative to healthy food; this is represented in the time trade-off balancing loop.

### Crime and social support

This feedback loop within the social stability and support theme (pink arrows) showed that when an individual is incarcerated as a result of a crime, ties within families and social networks are disrupted, reducing the number of stable homes and mentors in a community. One participant explained that a secure social support system is necessary to provide individuals, especially young people, with “spiritual grounding” for life skills, life goals, and awareness about one’s own health and the health of the community and environment. As stable homes and mentors diminish, individuals are more likely to be repeat offenders, and others in the community have a greater likelihood of participating in criminal activities, thus reinforcing the economic opportunity feedback loop. Further, participants linked family ties and mentors to greater knowledge of healthy food, reducing the perceived value of unhealthy food over healthy food, and increasing the purchasing of healthy food options.

### Action ideas

To close the workshop, the core modeling team elicited action ideas from the group to describe interventions to promote healthy food access. The team ranked these ideas along a matrix of difficulty of implementation and potential impact, with some suggestions falling within the “hard to do and high impact” and “easy to do and high impact” quadrants. These included efforts to build capacity in sourcing local produce in stores, support families in fostering a culture of healthy eating, increase knowledge of healthy foods particularly among caregivers, reduce crime and re-establish trust among community members including law enforcement, and engage schools to promote healthy eating behaviors (S3 Fig). Participants also spoke about the importance of community engagement and organizing in building wealth, as well as city policies and funding that can support the establishment of new stores, including those that stock healthy food options.

## Discussion

This is the first study to elicit and interrelate a range of factors contributing to the neighborhood food system and healthy food access in Baltimore, MD. The group model building workshop provided a means to generate dialogue between diverse stakeholders who normally do not have the opportunity to gather together, and to begin building capacity and self-efficacy for improving healthy food access in the community. The resulting synthesized causal loop diagram presented a framework to gain insight into the locally and contextually specific factors that influence food access in this urban environment, such as crime, economic opportunities, and social support. Findings also illustrate knowledge gaps to be further explored as well as potential intervention leverage points to be investigated through further modeling or natural experiments.

Our findings corroborate as well as extend prior research by providing a deeper and more nuanced framework for understanding the range and interconnectedness of barriers related to healthy food access. Participants described a predominance of unhealthy “chicken box stores” and little access to healthy food options, a theme echoed in former studies that have reported on greater access to unhealthier convenience and grocery stores and far fewer supermarkets in neighborhoods with higher levels of poverty [21,22]. At the store level, results associated with the challenges of stocking healthy food, such as consumer demand and profitability, were also confirmed by prior research [23,24]. The relationships between crime and several dimensions of the neighborhood food system present a unique and more comprehensive perspective than previous research by revealing the ways in which unhealthy food access is more viable than healthy food access in this context. One study reported on the relationships between urban crime and neighborhood food swamps [25]. However, much of the existing literature on crime and community health has focused on outcomes such as opportunities for and levels of physical activity and mental health [26,27]. To our knowledge, this is one of the first studies to report on the interconnected mechanisms through which crime impacts the neighborhood food system and healthy food access. For food businesses in the community, crime plays an interesting dual role in one sub-system by contributing to “income in the community” and reinforcing the “chicken box stores” feedback loop. These findings also share parallels with existing literature on alcohol outlets as crime generators in communities, confirming the need for future ethnographic studies to further understand the mechanisms and potential co-dependency between crime and establishments deemed less favorable for community health [28,29]. In addition, crime adds to the perceived riskiness, which may further discourage new and healthier stores from opening. Participants reflected on how efforts to reduce crime should consider the unintended consequences and differential impacts on community members. For example, removing crime may contribute to financial hardship for either the storeowner’s or drug dealer’s family due to a loss in income by forcing a store to go out of business or eliminating illicit drug trading, respectively. Finally, incarceration from criminal offenses impacts future employment opportunities and severs social ties, both of which negatively impact the offender and others in the social network including family members and friends, in terms of economic and educational opportunities that ultimately influence food behaviors. In future research, more quantitative and qualitative empirical evidence are needed to further build upon our understanding of urban food systems and strengthen strategies to improve healthy food access [25]. A critical part of this future work will be to elaborate on and address the root causes of crime, namely the social, economic, and environmental opportunities, and the extent to which they vary by place.

Outcomes from this study also shed new light on the complexity of healthy food perceived as “risky food,” due to the interplay of decisions made by both consumers and storeowners. Because many food stores in this context operate on small profit margins (e.g., independently owned corner stores, small grocers, and carryout restaurants), storeowners are primed to be risk-adverse, making less expensive, unhealthy food options more viable. Our causal loop diagram also depicts the complexity of healthy food purchasing for consumers, including available time for food preparation, marketing and advertising, and affordability. However, as one participant noted, not all healthy foods are equally “risky.” Even more, participants in our session shared concerns about the overabundance of “chicken box stores” and importantly, a real desire for greater variety in healthy food stores and healthy food options in the neighborhood. The nuanced insight into the role of “chicken box stores” and the conceptualization of “risky food” embedded in thorny feedback loops of community cohesion, crime, and health, argue for future research to better understand the degree of “riskiness” for a range of healthy food options, beyond typically stocked fresh produce with limited shelf lives. This further exploration may examine the economic costs and benefits to a “chicken box store” owner of stocking a larger variety of healthier food options, such as nuts, yogurt, and dried fruits should be investigated. Additionally, the extent to which the perceived riskiness of healthy food could be further reduced by investing in a more effective supply chain for such products as well as in marketing and awareness-building of such products is worth exploring.

The current qualitative model provides a contextually grounded framework for understanding dynamics of food access in this urban environment in the form of a causal loop diagram. This diagram offers a lens to explore potential leverage points that may take the form of a policy, program, or intervention. However, in order to build confidence in the model structure, and in order to test hypotheses of the relative impact of interventions acting on potential leverage points identified in the model, we would need to develop a more formal simulation model based on the structure identified in these workshop sessions. Future research would consist of building on the core structure of the synthesized causal loop diagram into a simulating stock and flow model. This next step would require the transformation of the synthesized model using the stock and flow grammar of system dynamics, either through another set of group model building workshops with community participants or through an alternative modeling process driven by a small group of experts, followed by community workshops to elicit critiques and suggested improvements to the model structure. Once a preliminary hypothesized structure is established, the logical consistency of the model could be tested through quantification of parameters and initial conditions, and the specification of the model structure as a system of coupled and ordinary differential equations [30]. This translation of a qualitative causal map to a formal quantitative simulation model allows for confidence building through identification of logical inconsistencies, tests of the model’s ability to replicate empirical data of historical trends, and identification of data gaps where quantitative data is not available for the specification of the model, as described in [31]. With greater confidence in the model, this formal simulation model would then allow researchers and policymakers to ask more complex “what if” questions about the potential impact of policy proposals and the relative leverage of various discrete and combined intervention strategies on healthy food access.

This research has some limitations. Findings that emerged are formative, generating refinements to theory development, new hypotheses, and potential policy interventions to be explored. Therefore, future research is needed to assess generalizability to other settings and populations. In addition, design choices related to the scripts employed during the workshop may have resulted in some perspectives being missed. Finally, the researcher perspective may influence the synthesis of the causal loop diagrams. Future studies could further test and refine the model and reduce researcher bias by conducting additional workshops in other neighborhoods of Baltimore, suburban communities, or another comparable city. Ethnographic studies and other qualitative inquiry to elaborate on the mechanisms proposed in this model, and primary data collection could be used to inform the development of a formal simulation model. Spatial analyses could also further corroborate our findings by quantitatively measuring the hypothesized relationships between the density of “chicken box stores” and crime activity in neighborhoods.

## Conclusions

A systems approach is valuable to provide a more comprehensive perspective of barriers to healthy food access, as well as illuminate the array of social, economic, and environmental mechanisms that relate to food access, by including multiple perspectives, identifying unexpected relationships, and understanding potential unintended consequences. In terms of the development of public health programs and policy, findings from this work contribute by: (1) establishing common language across multiple sectors about barriers related to healthy food access that may not have been explored previously; (2) contributing to the ongoing efforts of Reservoir Hill Improvement Council and Whitelock Community Farm by providing a new framework for improving healthy food access; and (3) laying the groundwork for building a simulation model based on the structure of the synthesized causal loop diagram, which could be used to explore stronger and weaker leverage points for creating change in the neighborhood food system.

## Supporting information

S1 Fig Participants working on Script #4, connection circles. (TIFF)

S2 Fig Participants working on Script #5, causal loop diagrams. (TIFF)

S3 Fig Potential intervention points (blue arrows) in the synthesized causal loop diagram to promote healthy food access. (TIFF)
