# Supplementary figures and images for: A community-based system dynamics approach suggests solutions for improving healthy food access in a low-income urban environment
Source: PLoS One. 2019 May 14;14(5):e0216985. doi: 10.1371/journal.pone.0216985 (PMC6516673; doi:10.1371/journal.pone.0216985)

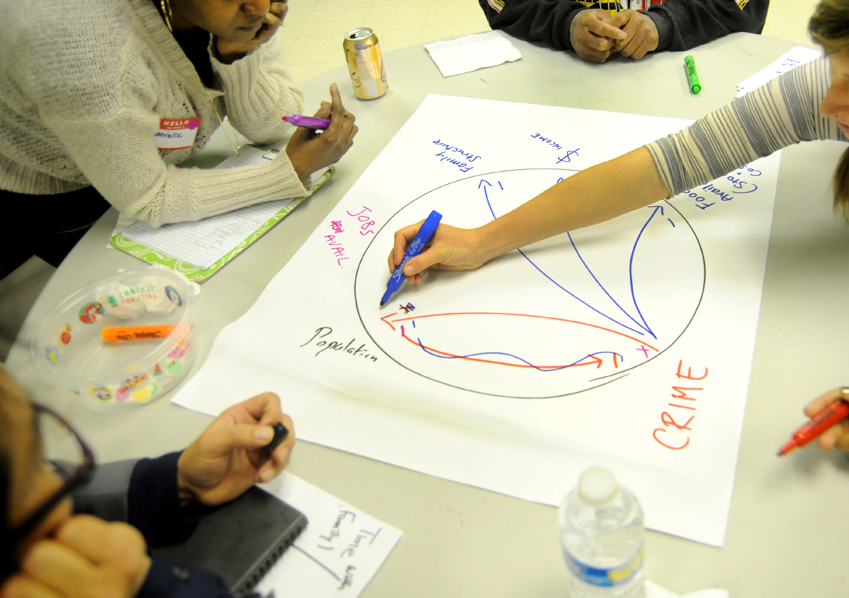

Supplement: S1 Fig — (TIFF) [file pone.0216985.s001.tiff]

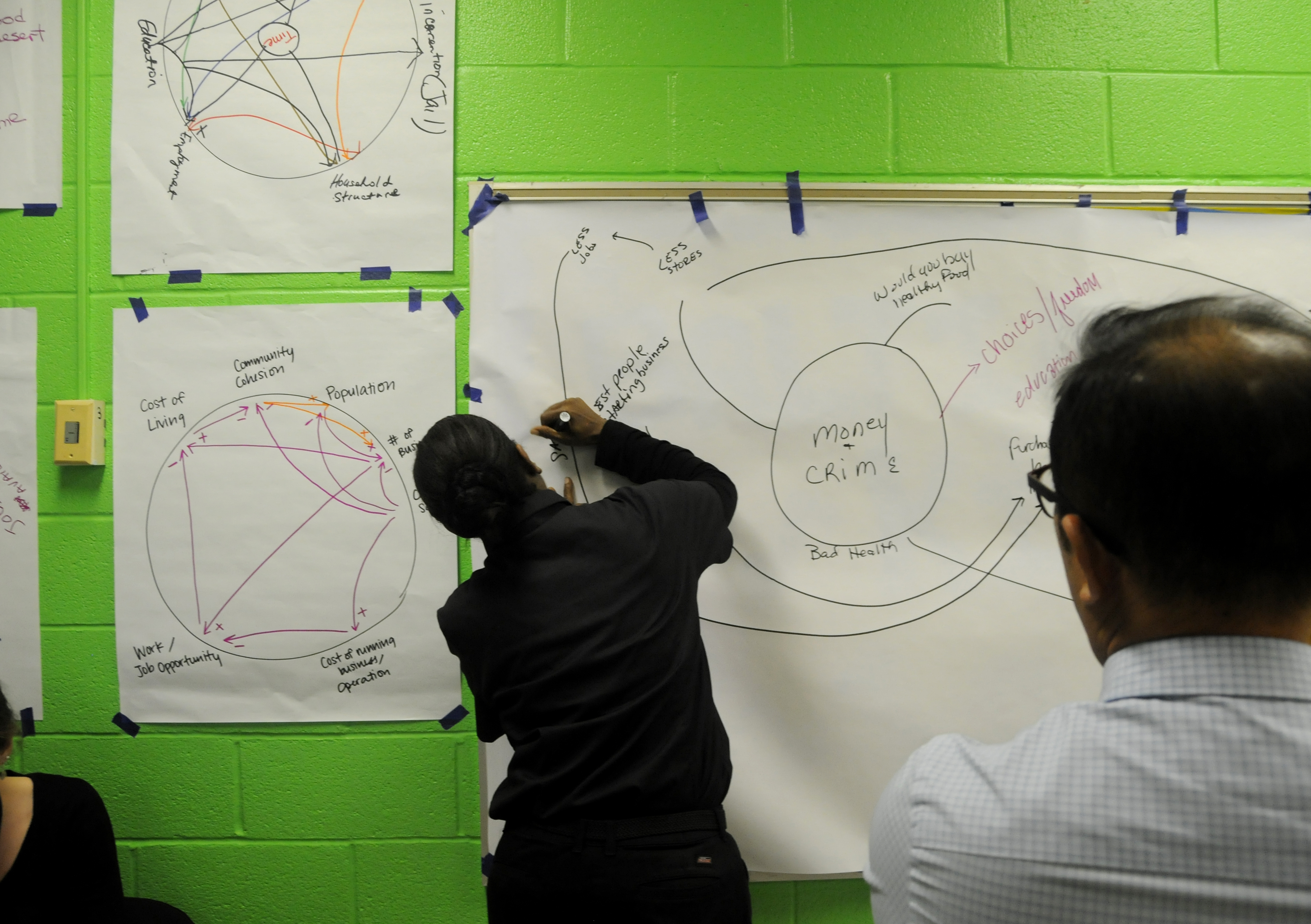

Supplement: S2 Fig — (TIFF) [file pone.0216985.s002.tiff]

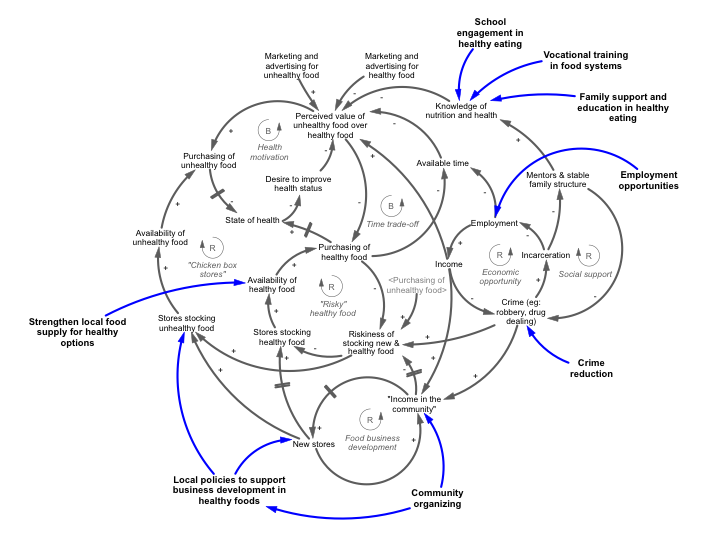

Supplement: S3 Fig — (TIFF) [file pone.0216985.s003.tiff]
